# Cutaneous Breast Cancer Metastasis Is Effectively Treated With Intralesional Interleukin-2 and Imiquimod: A Case Report and Brief Literature Review

**DOI:** 10.3389/fonc.2022.877014

**Published:** 2022-05-30

**Authors:** Ashley Drohan, Dejan Vidovic, Penelope Jane Barnes, Carman Anthony Giacomantonio, Lucy Kathryn Helyer

**Affiliations:** ^1^ Department of Surgery, University of Toronto, Toronto, ON, Canada; ^2^ Department of Surgery, Dalhousie University, Halifax, NS, Canada; ^3^ Department of Pathology, Dalhousie University, Halifax, NS, Canada

**Keywords:** cutaneous breast cancer, intralesional, interleukin-2 (IL-2), IL2, imiquimod, intralesional immunotherapy, intratumoral, cutaneous breast cancer metastasis

## Abstract

Breast cancer is the most common non-cutaneous cancer affecting women worldwide and is a major cause of cancer-related morbidity and mortality in females. While many women are diagnosed with early-stage disease, a subset of women may present with isolated cutaneous metastases or recurrent locoregional cutaneous metastatic disease. There is a paucity of evidence for effective treatments for cutaneous breast cancer metastases. Herein, we present a case of hormone receptor negative, HER2 positive cutaneous breast cancer metastasis treated with intralesional IL-2 and topical imiquimod, which was well tolerated with only minor low grade side effects. We also present a brief literature review of immunotherapy for cutaneous breast cancer metastasis to frame the discussion around using minimally invasive local therapies for this disease. Together, this limited data suggests that intralesional IL-2 and imiquimod may be considered as a safe option when treating a patient with cutaneous breast cancer metastases.

## Introduction

Breast cancer is the most common non-cutaneous cancer affecting women worldwide. Approximately 5% of patients present with *de novo* stage IV disease (1), with the most common sites of distant metastasis being bone, lung, and liver (2). Although cutaneous breast cancer metastases are rare (3), 69% of skin metastases among females with cancer are from a primary breast malignancy (4). While a minority of patients present with isolated cutaneous metastasis, most commonly they occur in the setting of distant metastatic disease. Currently, there is no well-defined treatment algorithm for cutaneous metastatic breast cancer.

Intralesional interleukin-2 (IL-2) injections have been used successfully to treat cutaneous metastases in other cancers, including melanoma (5) and porocarcinoma (6). However, the use of IL-2 in the treatment of breast cancer is not well-described, with a single case study published in 2021 reporting a pathologic complete response after intralesional IL-2 injections in a patient with triple-negative breast cutaneous cancer metastasis (7). Few others have had some success in treating cutaneous metastatic breast cancer with imiquimod, a topical Toll-like receptor 7 (TLR7) agonist (8–10). Herein, we describe the treatment of a patient with ER^-^/PR^-^ HER2^+^ cutaneous metastatic breast cancer using intralesional IL-2 and topical imiquimod to generate a durable complete clinical response.

## Case Description

### A Case of ER^-^/PR^-^ HER2^+^ Cutaneous Metastatic Breast Cancer

Our patient, a now 81-year-old female, was diagnosed with ER^-^/PR^-^ HER2^+^ left-sided breast cancer at the age of 54 (1994). She was initially treated with 6 cycles of neoadjuvant 5-fluorouracil, doxorubicin, cyclophosphamide (FAC), followed by lumpectomy, and adjuvant radiotherapy. Five years later, in 1999, she presented with a locoregional recurrence to the left chest wall and axilla, treated with a mastectomy, axillary dissection and adjuvant docetaxel, which was switched to 5-fluorouracil/folinic acid (FUFA) after two cycles due to disease progression. After a period of relative stability, at the age of 67 (2007), she was treated for a left chest wall recurrence with capecitabine and trastuzumab for a total of five years (2007-2012). Trastuzumab was discontinued within the first year of treatment (2007), due to cardiac toxicity. After experiencing stable disease for a period of five years, in 2012 surveillance computed tomography (CT) detected radiographic evidence of soft tissue metastases in the left chest wall, as well as axillary lymphadenopathy. Given the paucity of treatment options at that time, she was switched to vinorelbine. After one year of therapy, surveillance imaging demonstrated disease progression, and her regimen was then changed to paclitaxel with palliative intent. Additionally, she was treated with left upper chest wall radiotherapy. At the time, a repeat multigated acquisition (MUGA) scan revealed an ejection fraction (EF) of 65%; thus, the decision was made to start T-DM1. She remained on T-DM1 for only approximately 6 months, as it was ultimately stopped due to a significant decrease in EF. Additionally, she re-demonstrated disease progression with further development of contralateral (right) axillary lymphadenopathy that was treated with palliative radiotherapy to good effect. At this point, all systemic chemotherapeutic treatments were discontinued, and from 2015 to 2020 she was solely on zoledronic acid. She experienced a five-year period of relatively stable disease, with no further development of metastases on imaging.

**Figure 1:**
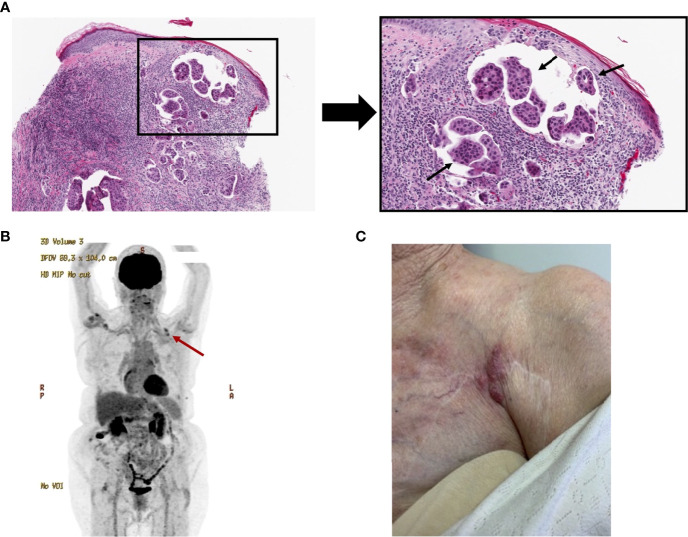
Initial presentation of an 81-year-old female with cutaneous breast cancer metastasis. **(A)** Histological H&E-stained image of cutaneous breast cancer metastasis, under 100X magnification (left) and higher power view, 200X magnification (right) of the metastatic adenocarcinoma, highlighted with arrows. This biopsy was obtained approximately at the first treatment timepoint. **(B)** PET-CT, obtained at the first treatment timepoint, revealing PET-avid uptake overlying the left pectoral muscle, highlighted with red arrow. **(C)** Image of the lesion pre-treatment.

After five years of stable disease, at the age of 79, she developed cutaneous lesions over the left chest wall. Excisional biopsy demonstrated viable adenocarcinoma consistent with her previous ER^-^/PR^-^ HER2^+^ metastatic breast cancer (**Supplementary Figure 1**), with a prominent chronic inflammatory infiltrate composed of lymphocytes and histiocytes (**Figure 1A**). Staging CT did not reveal any distant metastatic disease, and a positron emission tomography-computed tomography (PET-CT) scan demonstrated a solitary 12mm PET avid lesion on the left pectoral muscle, consistent with her physical exam findings (**Figures 1B, C**). Her case was reviewed by a multidisciplinary team, and it was decided to proceed with intralesional IL-2 treatment.

**Figure 2:**
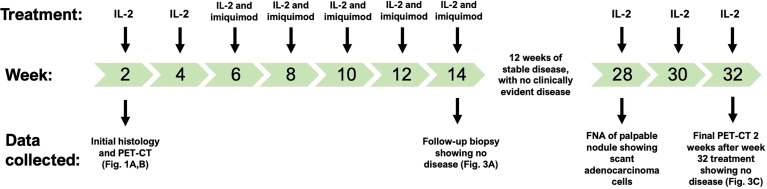
Treatment timeline for intralesional IL-2 injections and topical imiquimod. The patient was initially treated with IL-2 monotherapy, but showed only a partial response, after which imiquimod was added. The initial histology and PET-CT (**Figure 1**) were collected at week 2, the first treatment. Following 12 weeks of clinical stability with no evidence of disease, an FNA positive for adenocarcinoma led to three more IL-2 treatments, which lead to a complete clinical and radiological response (**Figure 3**).

Over the course of the next 14 weeks, she was treated with intralesional IL-2 every two weeks for a total of 7 treatments (**Figure 2**). Each treatment consisting of IL-2 contains 8 million international units (IU) per dose. At her third IL-2 treatment, physical exam revealed the chest wall nodule was now slightly more prominent, suggesting partial response to IL-2: thus, imiquimod cream was added to the biweekly IL-2 injections with the aim to promote and maintain a stronger anti-tumor immune response. Imiquimod is a TLR7 agonist and immunoadjuvant that has been used successfully for the treatment of in-transit melanoma and squamous cell carcinoma (11), and with which we have also had some success in treating primary cutaneous malignancies. It is dosed as daily topical application to the affected area for a total of five days, starting on the day of IL-2 injection. Our patient remained on biweekly IL-2 and imiquimod until the end of 14 weeks (**Figure 2**). Both treatments were very well tolerated, with side effects limited to grade 1/2 self-limiting fever, local injection site erythema, and fatigue all lasting less than 48 hours.

**Figure 3:**
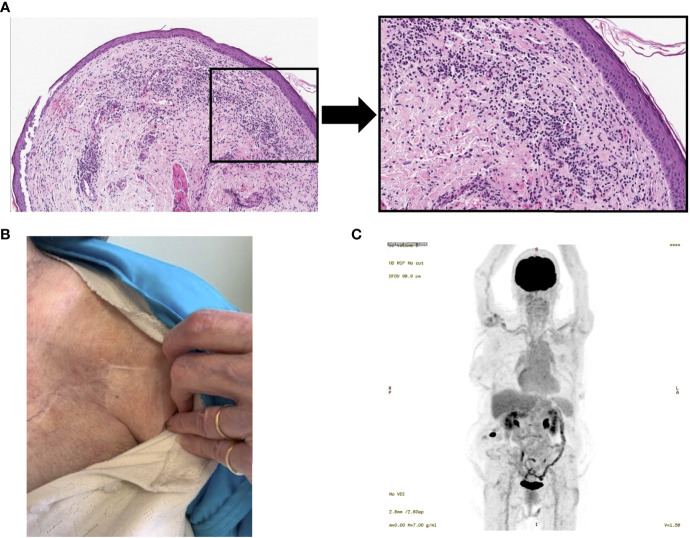
Resolution of cutaneous metastasis following intralesional IL-2 and topical imiquimod. **(A)** Punch biopsy of injected site at the 14-week timepoint, 100X magnification (left) and representative section, 200X magnification (right) showing no evidence of adenocarcinoma, with chronic immune infiltrates. **(B)** Image of lesion post-treatment, showing complete clinical response. **(C)** Post-treatment PET-CT (2 weeks after the final treatment; week 34) revealing no evidence of PET-avid lesions overlying the left pectoral muscle.

At the 14-week timepoint, a cutaneous chest wall punch biopsy was performed that revealed only a lymphocytic lichenoid infiltrate with vacuolar ulceration, and no evidence of metastatic or recurrent disease (**Figure 3A**). At the three-month follow-up, her physical exam revealed no evidence of cutaneous recurrence, however a palpable nodule was noted in the left flank. A CT scan revealed it was 10mm in size, and a fine-needle aspirate (FNA) demonstrated presence of scant adenocarcinoma cells in the specimen. She was treated with three intralesional IL-2 treatments, again dosed every two weeks. By then, the nodule had clinically completely resolved (**Figure 3B**), and her final PET-CT scan two weeks after the last injection revealed complete resolution of the left chest wall lesion and left flank (**Figure 3C**). At last follow-up, 24 weeks after completing therapy, she remained disease-free with no clinically evident lesions.

## Discussion

Herein, we present a case of ER^-^/PR^-^ HER2^+^ cutaneous metastatic breast cancer treated successfully using a local immunotherapy. Our patient underwent ten intralesional IL-2 treatments biweekly, and five topical imiquimod treatments for a five-day course starting on the third cycle of IL-2 treatments and continuing for five cycles, for a total of 32 weeks of treatment. Both therapies were tolerated very well with minor grade 1/2 adverse events. By the last follow-up 24 weeks after the last cycle of treatment, she had no clinical or radiological evidence of disease. This represents for the first time, to our knowledge, the treatment of cutaneous breast cancer metastasis with intralesional IL-2 and topical imiquimod immunotherapy.

In the last decade, there has been interest in the use of immunotherapies to treat cancer. Numerous immunotherapeutic agents have been trialed in many different cancers, such as chimeric antigen receptor T cell (CAR-T cell) therapies, immune checkpoint inhibitors, oncolytic viruses, and tumor vaccines. Immunotherapy in breast cancer is also an actively evolving field, illustrated by the recent FDA approval of pembrolizumab (a PD-1 inhibitor) (12) to be used as both a neoadjuvant therapy in combination with chemotherapy, or as adjuvant treatment alone for high-risk early stage triple-negative breast cancer (TNBC).

A significant challenge in the development and prospect of immunotherapy being used in breast cancer is the notion that compared to other cancers like melanoma or non-small cell lung cancer, breast cancer is predominantly immunologically quiescent (13). There are several steps required for the generation of anti-tumor immune responses, including adequate T cell trafficking into tumors, immunogenic cell death with neoantigen generation, and adequate neoantigen presentation. All or some are required to generate robust and potentially systemic anti-tumor immunity (14). Certainly, breast carcinomas that have a high mutational burden lend to having higher response rates and progression-free survival following immunotherapy. This is thought to be due to improved neoantigen stimulation of the immune system (15).

Within the landscape of breast cancer, there is evidence that ER^-^/PR^-^ HER2+ tumors (i.e. the biomarker profile of our patient’s disease) have higher mutational burden than hormone receptor positive tumors (16, 17), which is consistent with the observed clinical trial data leading to approval of pembrolizumab. In addition, approximately only 11% of breast cancers have a lymphocyte-predominant phenotype (greater than 50% tumor infiltrating lymphocytes, TILs) (18). HER2^+^ and TNBC tumors have the highest proportion of a lymphocyte-predominant phenotype (18). Clinically, patients with HER2^+^ cancers with higher percentages of TILs have a higher percentage of pathological complete responses following neoadjuvant trastuzumab and lapatinib, compared to HER2- patients (19). Our patient had histopathology suggestive of a significant inflammatory infiltrate, which may have aided in the generation of a complete response.

There is a paucity of data examining the immune microenvironment of cutaneous breast cancer metastases. However, other primary cutaneous malignancies, such as melanoma and cutaneous squamous cell carcinoma (cSCC) are regularly treated with local and/or intralesional immunotherapies (5). For cutaneous metastatic breast cancer, some intralesional therapies have been attempted in the past to varying success. Intralesional interferon alpha (IFNa) and interferon gamma (IFNg) achieved between a 43-71% lesional complete response rate, with evidence of anti-tumor immune responses in noninjected lesions (20). On the other end of the spectrum, intralesional injection of adenoviral vectors encoding IL-2 was not able to generate any conventional clinical responses (21). Similarly, a recent preclinical trial using c-Met-targeted CAR-T cells injected intralesionally in patients with cutaneous breast cancer metastases did not generate any clinical responses (22). Of note, a recent phase II trial using topical imiquimod monotherapy for cutaneous breast cancer metastases achieved a 20% partial response rate, also revealing that the cytokine response in the tumor microenvironment was enriched with activated CD8^+^ and CD4^+^ T cells, with increased local anti-tumor cytokine expression (23). Similarly, another recent phase II trial using systemic paclitaxel combined with topical imiquimod for cutaneous metastatic breast cancer generated a 36% complete response rate and 72% overall response rate (10). Other, smaller case studies have reported similar favorable findings using imiquimod with some patients experiencing complete responses (24). Together, this data illustrates imiquimod may have a favorable use in this setting. Intralesional IL-2, on the other hand, has not widely been used in cutaneous metastatic breast cancer, outside of a recent case report in 2021 (7). Certainly, in our patient, intralesional IL-2 monotherapy was able to generate a partial response initially. It was not until the addition of imiquimod that our patient experienced a complete response, which was then bolstered with additional IL-2 injections to a durable complete response.

Certainly, the use of both drugs led to a synergistic response in our patient. While the exact mechanism of action of these two drugs in this context has not been extensively studied, we hypothesize that the IL-2 likely provided a short-lived pulse of T cell-mediated anti-tumor immune activity, and the addition of imiquimod synergized the response further by acting as a strong local immune activator *via* TLR7 stimulation. Together, the two drugs were able to overcome immune quiescence through active stimulation of both arms of the immune system. In doing so, a robust anti-tumor immunity was mounted which led to tumor clearance.

In conclusion, our case has demonstrated the successful treatment of HER2+ cutaneous breast metastases with intralesional IL-2 and imiquimod. At present, our patient has tolerated 32 total weeks of treatment with minimal side effects and has no clinical or radiographic evidence of disease recurrence 24 weeks after her last treatment. These results suggest that intralesional IL-2 and topical imiquimod may be a durable treatment option among patients with cutaneous metastasis from breast cancers. While our patient experienced very minimal side effects, further study into safety profile of these drugs, especially in combination, is required. In addition, a potential drawback of this approach for both practitioners and patients are the frequency and duration of treatments. As this is a study of only a single case, further research to fully investigate this approach is required.

## Data Availability Statement

The original contributions presented in the study are included in the article/**Supplementary Material**. Further inquiries can be directed to the corresponding author.

## Ethics Statement

Ethical review and approval was not required for the study on human participants in accordance with the local legislation and institutional requirements. The patients/participants provided their written informed consent to participate in this study. Written informed consent was obtained from the individual(s) for the publication of any potentially identifiable images or data included in this article.

## Author Contributions

AD, DV, and LH contributed to data collection, project conception, and design of this manuscript and wrote sections of the manuscript. LH also contributed to project funding. PB contributed to data collection and manuscript editing. CG contributed to project design and manuscript editing. All authors contributed to the article and approved the submitted version.

## Conflict of Interest

The authors declare that the research was conducted in the absence of any commercial or financial relationships that could be construed as a potential conflict of interest.

## Publisher’s Note

All claims expressed in this article are solely those of the authors and do not necessarily represent those of their affiliated organizations, or those of the publisher, the editors and the reviewers. Any product that may be evaluated in this article, or claim that may be made by its manufacturer, is not guaranteed or endorsed by the publisher.

## Supplementary Material

Supplementary Figure 1 Excisional biopsy of local recurrence showing HER2 positive (3+) immunohistochemical staining of a subcutaneous tumor deposit, 100X magnification (left) and 200X magnification (right).
