# Supplementary figures and images for: Cutaneous Breast Cancer Metastasis Is Effectively Treated With Intralesional Interleukin-2 and Imiquimod: A Case Report and Brief Literature Review
Source: Front Oncol. 2022 May 30;12:877014. doi: 10.3389/fonc.2022.877014 (PMC9192334; doi:10.3389/fonc.2022.877014)

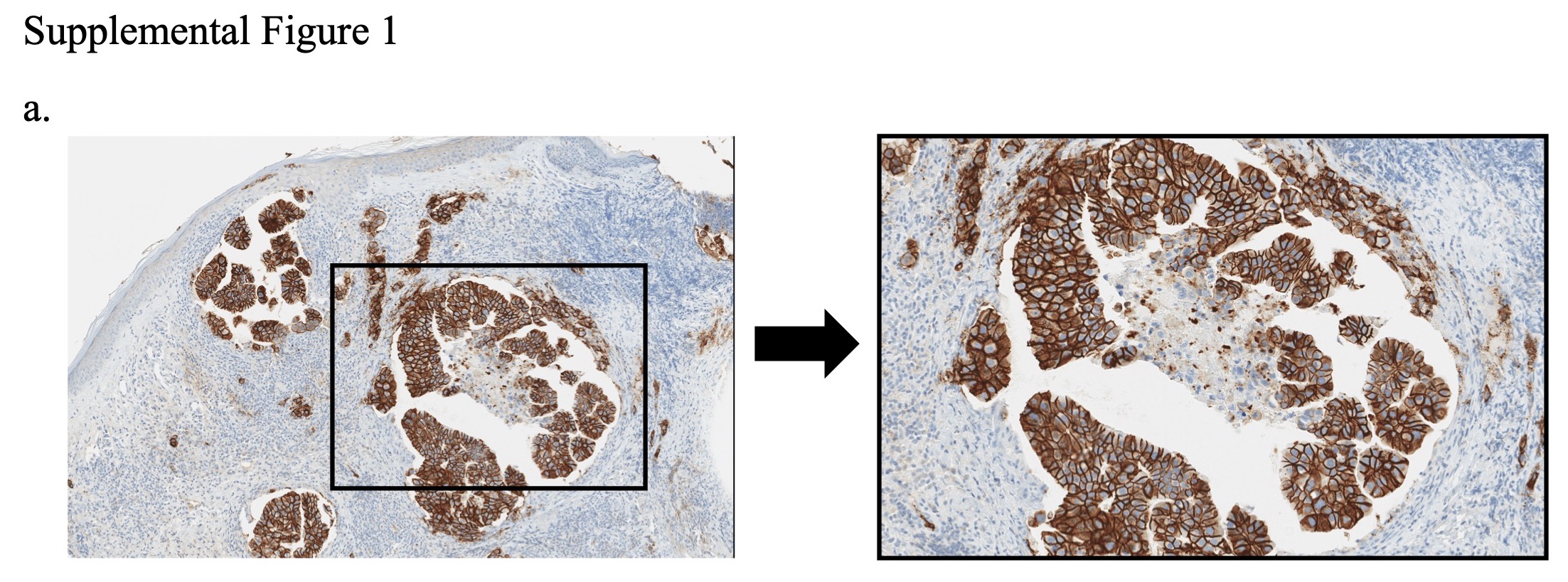

Supplement: Supplementary Figure 1 — Excisional biopsy of local recurrence showing HER2 positive (3+) immunohistochemical staining of a subcutaneous tumor deposit, 100X magnification (left) and 200X magnification (right). [file Image_1.jpeg]
